# [(3R*,4R*,5R*)-2,3-Diphenyl­isoxazolidine-4,5-di­yl]dimethanol

**DOI:** 10.1107/S1600536812025032

**Published:** 2012-06-13

**Authors:** Selahaddin Guner, Kűbra Şeftalicioglu

**Affiliations:** a Kocaeli University, Faculty of Art and Science, Department of Chemistry, 41380, Kocaeli, Turkey

## Abstract

In the title compound, C_17_H_19_NO_3_, the isoxazolidine ring adopts an envelope conformation with the O atom as the flap. In the crystal, O—H⋯O hydrogen bonds form *C*_2_^3^(14) *R*_2_^2^(14) motifs.

## Related literature

For general background to the preparation and use of compounds containing isoxazolidine rings, see: Agirbas *et al.* (2007 ▶); Kelly *et al.* (2009 ▶); Kumar *et al.* (2003 ▶); Kwon *et al.* (1995 ▶); Simonsen *et al.* (1999 ▶). For graph-set analysis of hydrogen-bonded networks, see: Bernstein *et al.* (1995 ▶). For ring conformations, see: Cremer & Pople (1975 ▶). For an alternative synthesis of the title compound, see: Tyukhteneva & Badovskaya (1992 ▶).
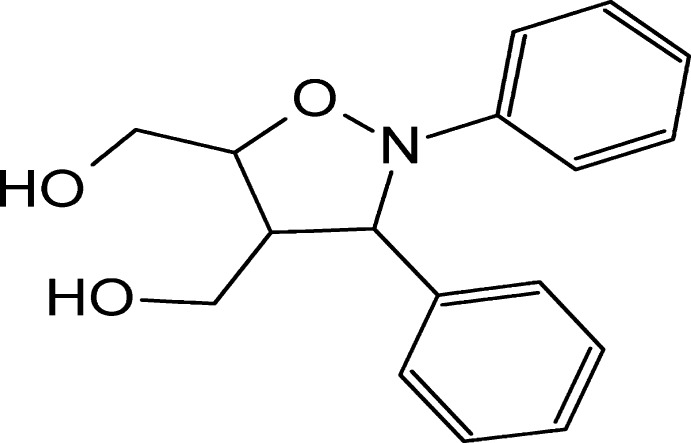


## Experimental

### 

#### Crystal data

C_17_H_19_NO_3_
*M* *_r_* = 285.33
Orthorhombic, 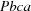

*a* = 8.1254 (2) Å
*b* = 11.0602 (2) Å
*c* = 32.4813 (10) Å
*V* = 2919.05 (13) Å^3^
*Z* = 8
Mo *K*α radiation
μ = 0.09 mm^−1^
*T* = 296 K
0.68 × 0.28 × 0.03 mm

#### Data collection

Stoe IPDS 2 diffractometer
Absorption correction: integration (*X-RED32*; Stoe & Cie, 2002 ▶) *T*_min_ = 0.985, *T*_max_ = 0.997
35240 measured reflections
2687 independent reflections
1915 reflections with *I* > 2σ(*I*)
*R*_int_ = 0.070

#### Refinement

*R*[*F* ^2^ > 2σ(*F* ^2^)] = 0.056
*wR*(*F* ^2^) = 0.108
*S* = 1.12
2687 reflections
198 parameters
H atoms treated by a mixture of independent and constrained refinement
Δρ_max_ = 0.12 e Å^−3^
Δρ_min_ = −0.14 e Å^−3^

### 

Data collection: *X-AREA* (Stoe & Cie, 2002 ▶); cell refinement: *X-AREA*; data reduction: *X-RED32* (Stoe & Cie, 2002 ▶); program(s) used to solve structure: *SHELXS97* (Sheldrick, 2008 ▶); program(s) used to refine structure: *SHELXL97* (Sheldrick, 2008 ▶); molecular graphics: *ORTEP-3 for Windows* (Farrugia, 1997 ▶); software used to prepare material for publication: *WinGX* (Farrugia, 1999 ▶).

## Supplementary Material

Crystal structure: contains datablock(s) I, global. DOI: 10.1107/S1600536812025032/ez2294sup1.cif

Supplementary material file. DOI: 10.1107/S1600536812025032/ez2294Isup2.mol

Structure factors: contains datablock(s) I. DOI: 10.1107/S1600536812025032/ez2294Isup3.hkl

Supplementary material file. DOI: 10.1107/S1600536812025032/ez2294Isup4.cml

Additional supplementary materials:  crystallographic information; 3D view; checkCIF report

## Figures and Tables

**Table 1:** Hydrogen-bond geometry (Å, °)

*D*—H⋯*A*	*D*—H	H⋯*A*	*D*⋯*A*	*D*—H⋯*A*
O2—H2*A*⋯O3^i^	0.86 (3)	1.90 (3)	2.756 (2)	172 (3)
O3—H3*A*⋯O2^ii^	0.86 (3)	1.91 (3)	2.738 (2)	160 (3)

Symmetry codes: (i) 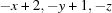; (ii) 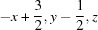.

**Fig. 1.**
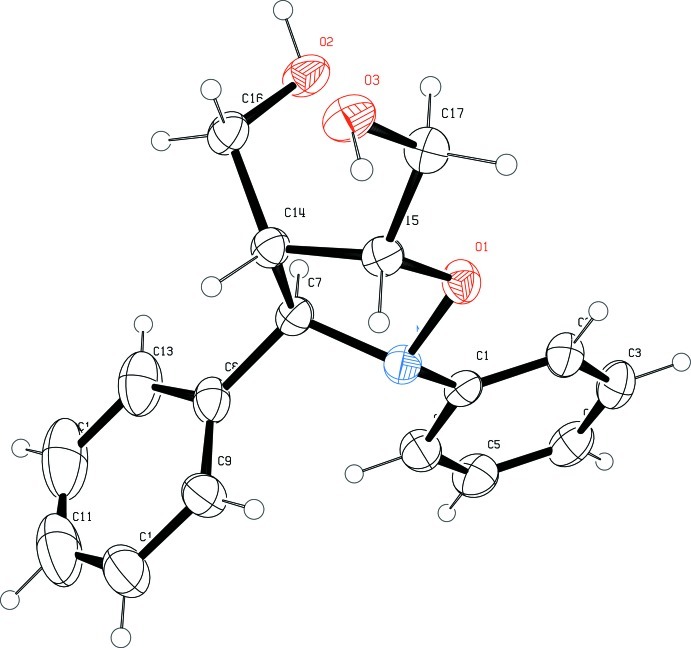
A view of the title structure with the atomic numbering scheme. Displacement ellipsoids are drawn at the 30% probability level.

**Fig. 2.**
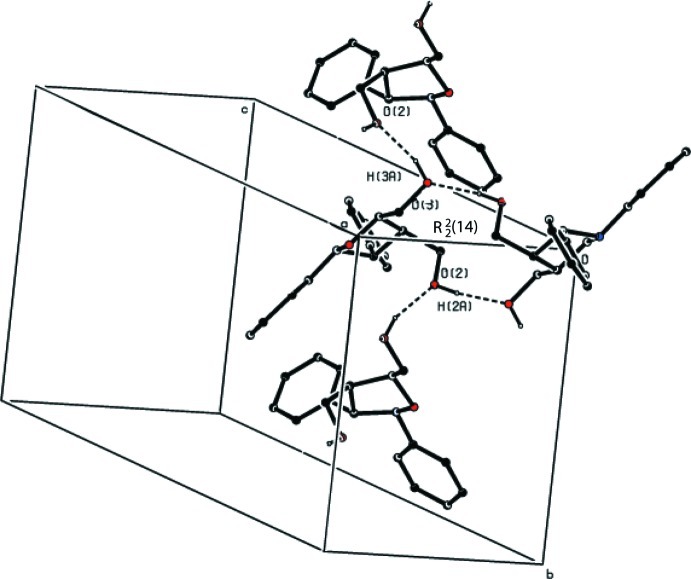
Part of the crystal structure of (I), showing the formation of hydrogen-bonded C23(14) R22(14) motifs. H atoms not involved in hydrogen bonds have been omitted for clarity. Dashed lines indicate hydrogen bonds. [Symmetry codes: (i) -x+2, -y+1, -z; (ii) -x+3/2, y-1/2, z].

## supplementary crystallographic information

### Comment

1,3-Dipolar cycloaddition reaction of nitrones are the best templates for the construction of isoxazolidine rings (Simonsen *et al.*, 1999). In recent years, useful anti-inflammatory (Kwon *et al.*, 1995), immunosuppressive and antibacterial (Kumar *et al.*, 2003) properties have been ascribed to molecules possessing such heterocyclic functionalities. In our previous work isoxazolidines obtained by 1,3-dipolar cycloaddition reactions have been found to have bioactivities to *Enterococcus faecalis* (ATCC 29212) and *Staphylococcus aureus* (ATCC 25923) (Agirbas *et al.*, 2007). A hydroxymethyl substituted isoxazolidine ring derivative was used as inhibitor for Human Purine Nucleoside Phosphorylase (PNP) (Kelly *et al.*, 2009). Furthermore, *cis*-2-butene-1,4-diol is used in the production of pharmaceuticals, plant-protection agents and pesticides. A previous report describes the preparation of the title compound (Tyukhteneva & Badovskaya, 1992), however, to the best of our knowledge there has been no study on the cycloaddition reaction of *C*,*N*-diphenylnitrone to *cis*-2-butene-1,4-diol. Therefore, we report herein the crystal structure of the title compound.

A perspective view of compound (I) with the atom-labelling scheme is shown in Fig. 1. The oxazolidine ring (O1/N1/C7/C14/C15) adopts an envelope conformation, with atom O1 displaced by 0.296 (1) Å from the other ring atoms (Cremer & Pople, 1975).

The crystal packing is stabilized by intermolecular O —H···O hydrogen bonds (Table 1). Fig. 2 shows that hydrogen bonds form *R*_2_^2^(14) motifs.

### Experimental

For the preparation of the title compound, *C*,*N*-diphenylnitrone (1 eq.) and *cis*-2-butene-1,4-diol (1.2 eq.) in 1-butanol:xylene (50:50) solvent mixture. This solution was heated and refluxed and monitored by TLC until all nitrone reacted. The solvent mixture was evaporated under vacuum. Residue was separated by using column chromatography, using a mixture of hexane-ethyl acetate (1:1) as the eluent. The product was a mixture of diastereomers. Recrystallization of the diastereomeric mixture in diethyl ether yielded only the *trans*-isomer single-crystal (m.p. 128.8 °C).

### Refinement

H atoms bonded to C atoms were positioned geometrically, with C—H = 0.93–0.98 Å and constrained to ride on their parent atoms [*U*_iso_(H) = 1.2*U*_eq_(C)]. Coordinates of O-bonded H atoms and O—H distances (0.86 Å) were refined freely [*U*_iso_(H)=1.5*U*_eq_(O)].

### Crystal data

**Table:**

C_17_H_19_NO_3_	*F*(000) = 1216
*M**_r_* = 285.33	*D*_x_ = 1.299 Mg m^−^^3^
Orthorhombic, *P**b**c**a*	Mo *K*α radiation, λ = 0.71073 Å
Hall symbol: -P 2ac 2ab	Cell parameters from 25949 reflections
*a* = 8.1254 (2) Å	θ = 1.3–26.2°
*b* = 11.0602 (2) Å	µ = 0.09 mm^−^^1^
*c* = 32.4813 (10) Å	*T* = 296 K
*V* = 2919.05 (13) Å^3^	Plate, colourless
*Z* = 8	0.68 × 0.28 × 0.03 mm

### Data collection

**Table:**

Stoe IPDS 2 diffractometer	2687 independent reflections
Radiation source: fine-focus sealed tube	1915 reflections with *I* > 2σ(*I*)
Graphite monochromator	*R*_int_ = 0.070
rotation method scans	θ_max_ = 25.6°, θ_min_ = 1.3°
Absorption correction: integration (*X-RED32*; Stoe & Cie, 2002)	*h* = −9→9
*T*_min_ = 0.985, *T*_max_ = 0.997	*k* = −12→12
35240 measured reflections	*l* = −39→39

### Refinement

**Table:**

Refinement on *F*^2^	Primary atom site location: structure-invariant direct methods
Least-squares matrix: full	Secondary atom site location: difference Fourier map
*R*[*F*^2^ > 2σ(*F*^2^)] = 0.056	Hydrogen site location: inferred from neighbouring sites
*wR*(*F*^2^) = 0.108	H atoms treated by a mixture of independent and constrained refinement
*S* = 1.12	*w* = 1/[σ^2^(*F*_o_^2^) + (0.0399*P*)^2^ + 0.523*P*] where *P* = (*F*_o_^2^ + 2*F*_c_^2^)/3
2687 reflections	(Δ/σ)_max_ < 0.001
198 parameters	Δρ_max_ = 0.12 e Å^−^^3^
0 restraints	Δρ_min_ = −0.14 e Å^−^^3^

### Special details

**Table:**

Geometry. All e.s.d.'s (except the e.s.d. in the dihedral angle between two l.s. planes) are estimated using the full covariance matrix. The cell e.s.d.'s are taken into account individually in the estimation of e.s.d.'s in distances, angles and torsion angles; correlations between e.s.d.'s in cell parameters are only used when they are defined by crystal symmetry. An approximate (isotropic) treatment of cell e.s.d.'s is used for estimating e.s.d.'s involving l.s. planes.
Refinement. Refinement of *F*^2^ against ALL reflections. The weighted *R*-factor *wR* and goodness of fit *S* are based on *F*^2^, conventional *R*-factors *R* are based on *F*, with *F* set to zero for negative *F*^2^. The threshold expression of *F*^2^ > σ(*F*^2^) is used only for calculating *R*-factors(gt) *etc*. and is not relevant to the choice of reflections for refinement. *R*-factors based on *F*^2^ are statistically about twice as large as those based on *F*, and *R*- factors based on ALL data will be even larger.

### Fractional atomic coordinates and isotropic or equivalent isotropic displacement parameters (Å^2^)

**Table:**

	*x*	*y*	*z*	*U*_iso_*/*U*_eq_	
C1	0.4711 (2)	0.6940 (2)	0.11533 (6)	0.0398 (5)	
C2	0.3582 (3)	0.7318 (2)	0.08617 (7)	0.0503 (6)	
H2	0.3525	0.6929	0.0608	0.060*	
C3	0.2538 (3)	0.8275 (2)	0.09473 (8)	0.0592 (7)	
H3	0.1788	0.8526	0.0749	0.071*	
C4	0.2587 (3)	0.8860 (2)	0.13183 (8)	0.0616 (7)	
H4	0.1872	0.9496	0.1374	0.074*	
C5	0.3716 (3)	0.8488 (3)	0.16085 (8)	0.0610 (7)	
H5	0.3774	0.8885	0.1860	0.073*	
C6	0.4761 (3)	0.7535 (2)	0.15292 (7)	0.0540 (6)	
H6	0.5507	0.7288	0.1730	0.065*	
C7	0.7547 (2)	0.6067 (2)	0.11543 (6)	0.0409 (5)	
H7	0.7889	0.6870	0.1060	0.049*	
C8	0.8016 (3)	0.5906 (2)	0.16002 (7)	0.0511 (6)	
C9	0.7355 (3)	0.4976 (3)	0.18331 (7)	0.0678 (8)	
H9	0.6590	0.4456	0.1715	0.081*	
C10	0.7812 (4)	0.4808 (4)	0.22366 (9)	0.1015 (13)	
H10	0.7354	0.4182	0.2390	0.122*	
C11	0.8926 (6)	0.5553 (6)	0.24095 (12)	0.129 (2)	
H11	0.9216	0.5439	0.2684	0.154*	
C12	0.9637 (5)	0.6469 (5)	0.21928 (15)	0.1221 (17)	
H12	1.0413	0.6969	0.2316	0.147*	
C13	0.9181 (4)	0.6648 (3)	0.17780 (10)	0.0838 (10)	
H13	0.9664	0.7264	0.1625	0.101*	
C14	0.8311 (2)	0.50842 (19)	0.08718 (6)	0.0397 (5)	
H14	0.8735	0.4437	0.1049	0.048*	
C15	0.6801 (2)	0.4591 (2)	0.06423 (6)	0.0401 (5)	
H15	0.6370	0.3897	0.0796	0.048*	
C16	0.9726 (2)	0.5551 (2)	0.06155 (7)	0.0461 (6)	
H16A	1.0594	0.5836	0.0796	0.055*	
H16B	1.0168	0.4896	0.0450	0.055*	
C17	0.6998 (3)	0.4225 (2)	0.01966 (6)	0.0458 (5)	
H17A	0.5934	0.4004	0.0084	0.055*	
H17B	0.7420	0.4903	0.0039	0.055*	
N1	0.5745 (2)	0.59162 (16)	0.10899 (5)	0.0400 (4)	
O1	0.56191 (16)	0.55449 (14)	0.06604 (4)	0.0442 (4)	
O2	0.9219 (2)	0.65171 (15)	0.03502 (5)	0.0548 (5)	
O3	0.80978 (19)	0.32278 (16)	0.01616 (5)	0.0491 (4)	
H2A	1.003 (4)	0.667 (3)	0.0184 (9)	0.097 (11)*	
H3A	0.757 (4)	0.260 (3)	0.0243 (9)	0.085 (10)*	

### Atomic displacement parameters (Å^2^)

**Table:**

	*U*^11^	*U*^22^	*U*^33^	*U*^12^	*U*^13^	*U*^23^
C1	0.0352 (10)	0.0386 (14)	0.0455 (11)	−0.0017 (9)	0.0046 (9)	0.0017 (10)
C2	0.0427 (11)	0.0530 (17)	0.0551 (13)	0.0039 (11)	−0.0043 (10)	−0.0042 (11)
C3	0.0432 (12)	0.0589 (18)	0.0756 (16)	0.0089 (12)	−0.0054 (12)	0.0019 (14)
C4	0.0475 (13)	0.0519 (17)	0.0855 (18)	0.0102 (12)	0.0134 (14)	−0.0037 (14)
C5	0.0607 (15)	0.0613 (19)	0.0610 (15)	0.0087 (13)	0.0095 (12)	−0.0122 (13)
C6	0.0541 (13)	0.0589 (18)	0.0489 (13)	0.0113 (12)	0.0017 (10)	−0.0040 (11)
C7	0.0357 (10)	0.0413 (13)	0.0457 (11)	−0.0019 (10)	0.0012 (9)	0.0007 (9)
C8	0.0432 (12)	0.0625 (18)	0.0477 (13)	0.0136 (12)	−0.0061 (10)	−0.0121 (11)
C9	0.0616 (15)	0.095 (2)	0.0466 (13)	0.0191 (15)	0.0036 (12)	0.0123 (14)
C10	0.082 (2)	0.170 (4)	0.0530 (17)	0.049 (2)	0.0043 (16)	0.020 (2)
C11	0.107 (3)	0.215 (6)	0.064 (2)	0.074 (4)	−0.028 (2)	−0.031 (3)
C12	0.098 (3)	0.153 (5)	0.114 (3)	0.033 (3)	−0.057 (3)	−0.064 (3)
C13	0.0696 (18)	0.091 (3)	0.091 (2)	0.0083 (17)	−0.0275 (16)	−0.0294 (18)
C14	0.0380 (10)	0.0374 (14)	0.0437 (11)	0.0024 (9)	0.0006 (9)	0.0026 (9)
C15	0.0410 (11)	0.0359 (13)	0.0433 (11)	0.0013 (10)	0.0031 (9)	0.0018 (9)
C16	0.0386 (11)	0.0431 (15)	0.0565 (13)	0.0047 (10)	0.0041 (10)	0.0040 (11)
C17	0.0473 (12)	0.0415 (15)	0.0485 (12)	0.0009 (10)	−0.0002 (9)	−0.0008 (10)
N1	0.0380 (9)	0.0433 (12)	0.0387 (9)	0.0015 (8)	−0.0003 (7)	−0.0041 (8)
O1	0.0426 (8)	0.0473 (10)	0.0427 (8)	0.0069 (7)	−0.0046 (6)	−0.0064 (7)
O2	0.0480 (9)	0.0482 (11)	0.0681 (10)	0.0066 (8)	0.0145 (8)	0.0167 (8)
O3	0.0481 (9)	0.0402 (11)	0.0590 (10)	0.0006 (8)	0.0111 (7)	−0.0021 (8)

### Geometric parameters (Å, º)

**Table:**

C1—C2	1.383 (3)	C10—H10	0.9300
C1—C6	1.388 (3)	C11—C12	1.362 (6)
C1—N1	1.425 (3)	C11—H11	0.9300
C2—C3	1.385 (3)	C12—C13	1.411 (5)
C2—H2	0.9300	C12—H12	0.9300
C3—C4	1.368 (3)	C13—H13	0.9300
C3—H3	0.9300	C14—C16	1.511 (3)
C4—C5	1.378 (3)	C14—C15	1.536 (3)
C4—H4	0.9300	C14—H14	0.9800
C5—C6	1.378 (3)	C15—O1	1.428 (2)
C5—H5	0.9300	C15—C17	1.512 (3)
C6—H6	0.9300	C15—H15	0.9800
C7—N1	1.488 (3)	C16—O2	1.433 (3)
C7—C8	1.508 (3)	C16—H16A	0.9700
C7—C14	1.552 (3)	C16—H16B	0.9700
C7—H7	0.9800	C17—O3	1.424 (3)
C8—C13	1.380 (4)	C17—H17A	0.9700
C8—C9	1.385 (4)	C17—H17B	0.9700
C9—C10	1.375 (4)	N1—O1	1.458 (2)
C9—H9	0.9300	O2—H2A	0.86 (3)
C10—C11	1.347 (6)	O3—H3A	0.86 (3)
			
C2—C1—C6	118.6 (2)	C11—C12—C13	119.1 (4)
C2—C1—N1	122.14 (19)	C11—C12—H12	120.5
C6—C1—N1	119.14 (19)	C13—C12—H12	120.5
C1—C2—C3	120.0 (2)	C8—C13—C12	119.8 (4)
C1—C2—H2	120.0	C8—C13—H13	120.1
C3—C2—H2	120.0	C12—C13—H13	120.1
C4—C3—C2	121.4 (2)	C16—C14—C15	117.50 (17)
C4—C3—H3	119.3	C16—C14—C7	113.01 (18)
C2—C3—H3	119.3	C15—C14—C7	102.49 (15)
C3—C4—C5	118.7 (2)	C16—C14—H14	107.8
C3—C4—H4	120.6	C15—C14—H14	107.8
C5—C4—H4	120.6	C7—C14—H14	107.8
C6—C5—C4	120.7 (2)	O1—C15—C17	107.96 (16)
C6—C5—H5	119.6	O1—C15—C14	104.74 (16)
C4—C5—H5	119.6	C17—C15—C14	118.40 (17)
C5—C6—C1	120.6 (2)	O1—C15—H15	108.5
C5—C6—H6	119.7	C17—C15—H15	108.5
C1—C6—H6	119.7	C14—C15—H15	108.5
N1—C7—C8	111.72 (17)	O2—C16—C14	111.57 (17)
N1—C7—C14	103.43 (16)	O2—C16—H16A	109.3
C8—C7—C14	112.57 (18)	C14—C16—H16A	109.3
N1—C7—H7	109.7	O2—C16—H16B	109.3
C8—C7—H7	109.7	C14—C16—H16B	109.3
C14—C7—H7	109.7	H16A—C16—H16B	108.0
C13—C8—C9	118.6 (2)	O3—C17—C15	110.49 (17)
C13—C8—C7	120.3 (3)	O3—C17—H17A	109.6
C9—C8—C7	120.9 (2)	C15—C17—H17A	109.6
C10—C9—C8	121.1 (3)	O3—C17—H17B	109.6
C10—C9—H9	119.5	C15—C17—H17B	109.6
C8—C9—H9	119.5	H17A—C17—H17B	108.1
C11—C10—C9	119.8 (4)	C1—N1—O1	108.72 (15)
C11—C10—H10	120.1	C1—N1—C7	118.06 (17)
C9—C10—H10	120.1	O1—N1—C7	103.59 (13)
C10—C11—C12	121.7 (4)	C15—O1—N1	101.56 (13)
C10—C11—H11	119.2	C16—O2—H2A	107 (2)
C12—C11—H11	119.2	C17—O3—H3A	107 (2)
			
C6—C1—C2—C3	0.3 (3)	N1—C7—C14—C15	6.3 (2)
N1—C1—C2—C3	176.2 (2)	C8—C7—C14—C15	127.02 (19)
C1—C2—C3—C4	−0.4 (4)	C16—C14—C15—O1	−101.0 (2)
C2—C3—C4—C5	0.7 (4)	C7—C14—C15—O1	23.48 (19)
C3—C4—C5—C6	−0.9 (4)	C16—C14—C15—C17	19.3 (3)
C4—C5—C6—C1	0.9 (4)	C7—C14—C15—C17	143.79 (19)
C2—C1—C6—C5	−0.5 (4)	C15—C14—C16—O2	58.9 (3)
N1—C1—C6—C5	−176.6 (2)	C7—C14—C16—O2	−60.2 (2)
N1—C7—C8—C13	−139.9 (2)	O1—C15—C17—O3	−176.79 (16)
C14—C7—C8—C13	104.2 (3)	C14—C15—C17—O3	64.6 (3)
N1—C7—C8—C9	43.5 (3)	C2—C1—N1—O1	10.9 (3)
C14—C7—C8—C9	−72.4 (3)	C6—C1—N1—O1	−173.18 (19)
C13—C8—C9—C10	1.7 (4)	C2—C1—N1—C7	128.4 (2)
C7—C8—C9—C10	178.3 (2)	C6—C1—N1—C7	−55.7 (3)
C8—C9—C10—C11	−0.3 (5)	C8—C7—N1—C1	85.1 (2)
C9—C10—C11—C12	−0.9 (6)	C14—C7—N1—C1	−153.59 (17)
C10—C11—C12—C13	0.7 (7)	C8—C7—N1—O1	−154.71 (17)
C9—C8—C13—C12	−1.8 (4)	C14—C7—N1—O1	−33.39 (19)
C7—C8—C13—C12	−178.5 (3)	C17—C15—O1—N1	−171.89 (16)
C11—C12—C13—C8	0.7 (6)	C14—C15—O1—N1	−44.85 (17)
N1—C7—C14—C16	133.72 (17)	C1—N1—O1—C15	175.74 (15)
C8—C7—C14—C16	−105.5 (2)	C7—N1—O1—C15	49.38 (18)

### Hydrogen-bond geometry (Å, º)

**Table:**

*D*—H···*A*	*D*—H	H···*A*	*D*···*A*	*D*—H···*A*
O2—H2*A*···O3^i^	0.86 (3)	1.90 (3)	2.756 (2)	172 (3)
O3—H3*A*···O2^ii^	0.86 (3)	1.91 (3)	2.738 (2)	160 (3)

Symmetry codes: (i) −*x*+2, −*y*+1, −*z*; (ii) −*x*+3/2, *y*−1/2, *z*.
